# Parathyroidectomy Positively Modulates Systemic Inflammation and Nutritional Status: Immune-Inflammation Index and Prognostic Nutritional Index in Primary Hyperparathyroidism

**DOI:** 10.3390/medicina61071236

**Published:** 2025-07-08

**Authors:** Yusuf Karadeniz, Melia Karakose

**Affiliations:** 1 Department of Internal Medicine, Division of Endocrinology and Metabolism, Karaman Training and Research Hospital, Karaman 70200, Türkiye; 2 Department of Internal Medicine, Division of Endocrinology and Metabolism, Faculty of Medicine, Necmettin Erbakan University, Konya 42090, Türkiye; meliakarakose@yahoo.com

**Keywords:** hyperparathyroidism, primary, parathyroidectomy, systemic inflammation, prognostic nutritional index, C-reactive protein

## Abstract

*Background/Objectives*: Primary hyperparathyroidism (PHPT) has been associated with systemic inflammation and metabolic disturbances. This study aimed to evaluate changes in the Systemic Immune-Inflammation Index (SII) and Prognostic Nutritional Index (PNI) following parathyroidectomy (PTX) in PHPT patients, and to assess their return toward healthy control values. *Materials and Methods*: This retrospective study was conducted between January 2010 and March 2022. It analyzed the demographic characteristics, clinical findings, and laboratory results of patients diagnosed with and operated for PHPT, with comparisons to healthy controls. Postoperative values were recorded at least six months after surgery. Bone mineral density was classified according to World Health Organization criteria, and nephrolithiasis was assessed with imaging. Results: After applying exclusion criteria, 415 PHPT patients and 410 controls were included. PHPT patients were older (*p* < 0.001) and had a higher proportion of females (*p* = 0.016). Compared to controls, they had lower phosphorus, albumin, high-density lipoprotein cholesterol, total cholesterol, hemoglobin, and PNI (*p* < 0.001 for all), while triglycerides, monocytes, platelets, CRP, and SII were higher (*p* < 0.05). Postoperatively, albumin, platelets, total cholesterol, and triglycerides increased (*p* < 0.001), while calcium, white blood cell count, neutrophils, lymphocytes, and CRP decreased (*p* < 0.05), approaching healthy control values. In age- and sex-matched comparisons (propensity score matching, *n* = 259 in each group), platelets (*p* = 0.002) and hemoglobin (*p* = 0.018) were found to be higher postoperatively. *Conclusions*: Preoperative SII and PNI levels were significantly altered in PHPT patients compared to healthy controls. Following PTX, both of these markers and other parameters showed significant improvements, reflecting positive changes in systemic inflammation and nutritional status.

## 1. Introduction

Primary hyperparathyroidism (PHPT) is a disorder characterized by excessive secretion of parathyroid hormone (PTH), commonly leading to hypercalcemia [1]. The prevalence of PHPT in the general population ranges between 0.1% and 0.3%, with a relatively higher frequency in older age [1,2,3,4,5]. The most common cause of PHPT is parathyroid adenoma, while parathyroid hyperplasia or parathyroid carcinoma may also underlie the disease [1,2,3,4,5].

Clinically, PHPT presents with a broad spectrum, ranging from asymptomatic cases with mild hypercalcemia to those with severe metabolic complications [4]. PHPT has been associated with serious long-term health issues, including hypertension, insulin resistance, dyslipidemia, cardiovascular diseases, osteoporosis, and an increased incidence of cancer [1,5,6,7,8]. Patients with hypercalcemia can experience gastrointestinal disturbances, nephrolithiasis, decreased bone mineral density (BMD), and neuropsychiatric symptoms [1,2,3]. The main treatment for PHPT is surgery, and a successful parathyroidectomy (PTX) results in the improvement of biochemical parameters as well as an increase in bone mineral density [1,9,10].

Recent studies have suggested that PHPT may be directly and indirectly associated with inflammatory processes [11,12,13]. Hypercalcemia, alterations in phosphorus metabolism, and other effects of PTH may contribute to the activation of the inflammatory response [11,12,13]. In this context, various biochemical parameters assessing inflammation and nutritional status have been proposed as potential predictors of clinical outcomes in PHPT patients. The Prognostic Nutritional Index (PNI) is a parameter used to assess nutritional status and clinical prognosis, calculated based on serum albumin levels and lymphocyte count [14]. PNI has been reported as a prognostic indicator in malignancies and various chronic diseases [15,16,17]. The Systemic Immune-Inflammation Index (SII), which is calculated using lymphocyte, neutrophil, and platelet counts, serves as an inflammation-related biomarker [14]. Elevated SII levels have been associated with poor prognosis in a wide range of diseases, including cardiovascular disorders and malignancies [5,14,17,18]. However, data regarding preoperative and postoperative changes in PNI and SII in PHPT patients following PTX remain limited.

Our primary objective was to investigate the changes in SII and PNI values following PTX. Secondarily, we aimed to examine postoperative changes in other inflammatory and laboratory markers, while also performing comparisons with healthy controls.

## 2. Materials and Methods

### 2.1. Ethics, Design and Subjects

This study was designed in accordance with the expected ethical principles and was approved to be in compliance with the Declaration of Helsinki and its later amendments by the local ethics committee.

This was a retrospective cohort study conducted at the Department of Endocrinology, our university. This study included patients diagnosed with PHPT who underwent surgery between January 2010 and March 2022 (patient group) and healthy individuals who visited the outpatient clinic for other reasons and had complete data for the study variables (control group). The common exclusion criteria for both groups were as follows: being younger than 18 years of age, having metabolic or endocrine disorders (except hyperparathyroidism in the patient group), having incomplete data, presence of chronic diseases, parathyroid or any other organ malignancy, inflammatory or hematologic diseases, active infections, renal or hepatic insufficiency, heart failure, pregnancy or lactation, use of medications known to affect complete blood count indices, and use of the medications including statins, fibrates, corticosteroids, NSAIs. Additionally, PHPT patients who did not undergo surgery and patients with persistent PHPT were not included in the study. Patients with a known family history of hyperparathyroidism, jaw tumor syndrome, MEN syndromes, or early age at onset (<30 years) were assessed for potential genetic forms and excluded if suspected.

### 2.2. Data Collection

#### 2.2.1. Demographics and Diagnosis

The collected data included demographic characteristics, clinical features, and laboratory findings. Demographic variables included patients’ sex, age at PHPT diagnosis, and current age. Disease duration was calculated as the difference between the date of diagnosis and the last follow-up. For the control group, only the age at study inclusion was recorded. Laboratory parameters were obtained from the hospital information system, including values measured at the time of diagnosis and follow-up. Blood samples were collected during routine clinical practice, and biochemical analyses were conducted using standard methods in the hospital’s biochemistry laboratory.

The diagnosis of PHPT was established based on biochemical and clinical parameters [1,5]. Elevated serum PTH levels accompanied by hypercalcemia (serum calcium > 10.2 mg/dL), supported by increased 24 h urinary calcium excretion. Disease localization was determined through imaging modalities such as ultrasonography and/or parathyroid scintigraphy at diagnosis or intraoperative observations. If adenoma existed, localization was classified as follows: right inferior, right superior, left inferior, left superior, right intrathyroidal, left intrathyroidal, and mediastinal. Patients’ clinical conditions, nephrolithiasis status (presence/absence), and final status (alive/deceased) were recorded. Nephrolithiasis classification was based on imaging findings. These included renal ultrasonography or non-contrast computed tomography, as per routine clinical practice. Patients with a known family history of hyperparathyroidism, jaw tumor syndrome, MEN syndromes, or early age at onset (<30 years) were assessed for potential genetic forms and excluded if suspected.

#### 2.2.2. Laboratory Measurements

All measurements were performed via routine methods from standardized antecubital blood collection. For postoperative blood values, the latest available measurements obtained at least six months after surgery were examined. Both baseline and postoperative results from the PHPT group were compared to healthy controls. Appropriate paired analyses were also performed within the PHPT group, as allowed by the data.

Parameters quantified included serum calcium, phosphorus, PTH, 25-hydroxyvitamin D3 (25(OH)D3), creatinine, alkaline phosphatase (ALP), albumin, C-reactive protein (CRP), and lipid profile [total cholesterol, low-density lipoprotein cholesterol (LDL-C), and high-density lipoprotein cholesterol (HDL-C), triglycerides]. Complete blood count parameters [white blood cell (WBC) count, neutrophil count, lymphocyte count, monocyte count, hemoglobin, red cell distribution width (RDW), platelet count] were also recorded. PTH levels were measured using an electrochemiluminescence immunoassay method, with reference values set at 15–65 pg/mL. The reference ranges were 8.8–10.2 mg/dL for serum calcium, 2.5–4.5 mg/dL for phosphorus, and 35–50 g/L for albumin. Hypocalcemia was defined as a serum calcium level below 8.8 mg/dL [19]. Complete blood count parameters were analyzed using automated hematology analyzers. Lipid profile analyses were conducted using enzymatic colorimetric methods, with reference values as follows: total cholesterol < 200 mg/dL, LDL-C < 130 mg/dL, HDL-C ≥ 40 mg/dL, and triglycerides < 150 mg/dL.

All biochemical analyses were conducted using standardized automated methods. Internal quality control was performed daily and external quality control was ensured via national EQA programs.

#### 2.2.3. Inflammatory Indices

SII and PNI were determined from available data. SII was calculated using the following formula: SII = (platelet count × neutrophil count)/lymphocyte count. An increase in SII is considered indicative of heightened systemic inflammation [5]. PNI was calculated using the following formula: PNI = (albumin level × 10) + lymphocyte count (×103) [5]. Higher PNI values indicate good nutritional status and overall healthy metabolic condition [5]. MHR (monocyte to high-density lipoprotein ratio) was calculated as the ratio of monocyte count to HDL-C level [5].

#### 2.2.4. Bone Mineral Density

The BMD measurements were recorded as T-scores after measurements from the femoral neck, forearm, and lumbar spine regions. Osteoporosis classification was performed according to the World Health Organization (WHO) criteria [20]: (i) Normal: T-score ≥ −1.0; (ii)- Osteopenia: T-score between −1.1 and −2.4; (iii) Osteoporosis: T-score ≤ −2.5.

### 2.3. Endpoints

The primary endpoint of the study was the comparison of preoperative and postoperative SII and PNI values. Secondary endpoints included investigating preoperative to postoperative changes in other laboratory parameters, identifying factors associated with SII and PNI in PHPT patients, comparing data variables between patient and control groups and analyzing independent predictors of nephrolithiasis, osteoporosis, and postoperative hypocalcemia.

### 2.4. Statistical Analysis

All analyses were performed on IBM SPSS Statistics for Windows, Version 25.0 (IBM Corp., Armonk, NY, USA). Two-tailed *p*-values of less than 0.05 were accepted as statistically significant. Normality assumption was evaluated using visual inspection of histograms and Q-Q plots. Descriptive statistics were presented using the mean ± standard deviation for normally distributed continuous variables, median (25th percentile–75th percentile) for non-normally distributed continuous variables, and frequency (percentage) for categorical variables. Repeated measurements of continuous variables were analyzed using the paired *t*-test or Wilcoxon signed ranks test depending on normality of distribution. Pearson or Spearman correlation coefficients were calculated to evaluate relationships between continuous variables. Between-groups analyses of continuous variables were performed using the Student’s *t*-test or the Mann–Whitney U test, again depending on normality of distribution. Categorical variables were compared using appropriate chi-square tests. Propensity score matching was performed to match groups for sex and age, with additional comparisons based on these matched groups. Multivariable logistic regression analysis (forward conditional selection method) was performed to determine independent predictors of postoperative hypocalcemia. Variables were analyzed using the univariable logistic regression analysis and statistically significant variables were included to the multivariable logistic regression analysis. Pearson, Spearman, and point biserial coefficients were used to evaluate relationships between postoperative inflammation markers and other variables.

## 3. Results

**Figure 1:**
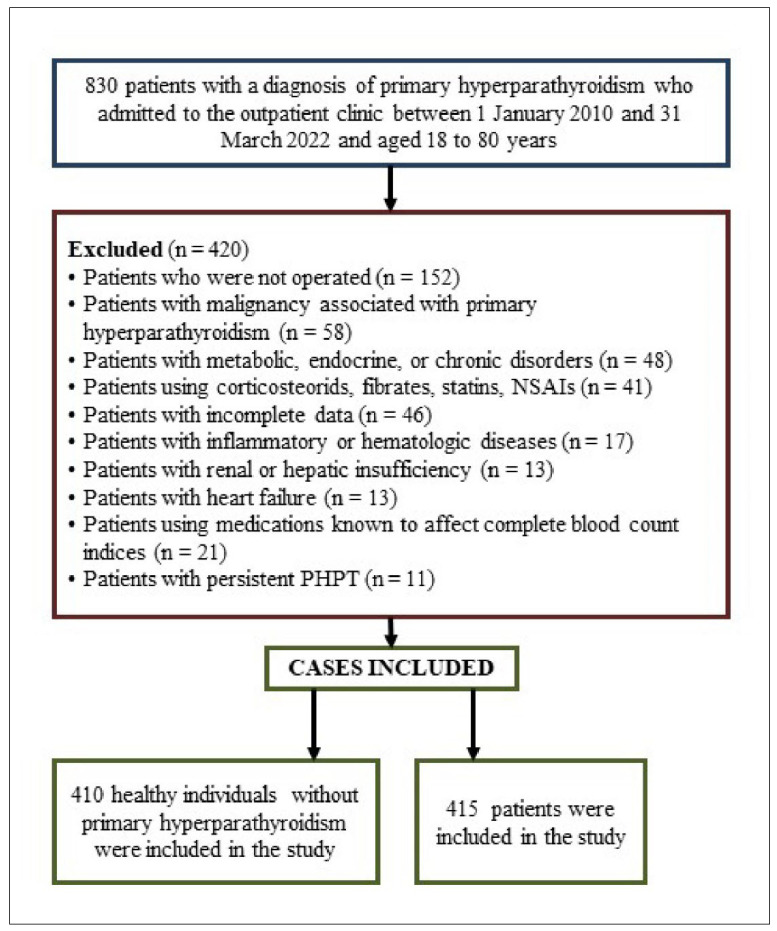
Flowchart of the study.

A total of 835 patients with PHPT were evaluated. After applying the exclusion criteria, 415 patients were included in the study. Additionally, 410 healthy controls were included in the study (Flowchart in Figure 1).

Of the patients, 80.24% (*n* = 333) were female, with a mean age at diagnosis of 53.4 ± 12.8 and a current mean age of 57.8 ± 13.3 years. The most common adenoma site was the right inferior quadrant (44.96%), followed by the left inferior quadrant (40.63%); the least common site was the left intrathyroidal region (0.29%). The demographic and disease-related characteristics of the patients are presented in Table 1.

Baseline and postoperative laboratory measurements of patients with PHPT are presented in Table 2. In patients with PHPT, albumin, platelet, total cholesterol, and triglyceride levels significantly increased postoperatively (*p* < 0.001 for all except platelet: *p* = 0.038). Conversely, calcium (*p* < 0.001), WBC (*p* = 0.019), neutrophil (*p* < 0.001), lymphocyte (*p* = 0.002), and CRP (*p* < 0.001) levels significantly decreased after surgery.

**Figure 2:**
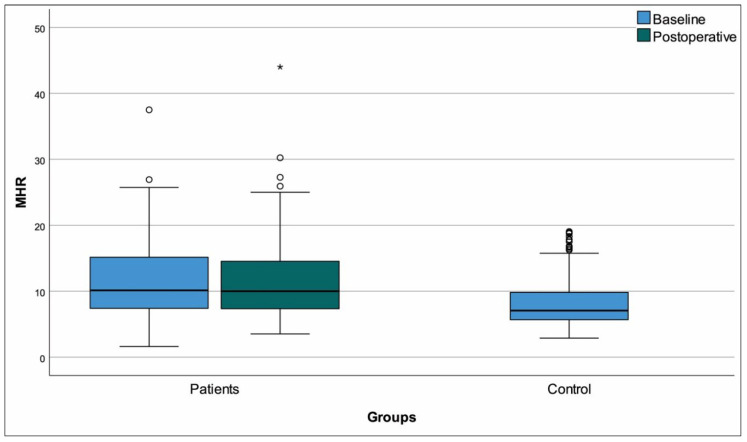
Box-plots monocyte to high-density lipoprotein cholesterol ratio. “o Outliers, * Extreme outliers”.

**Figure 3:**
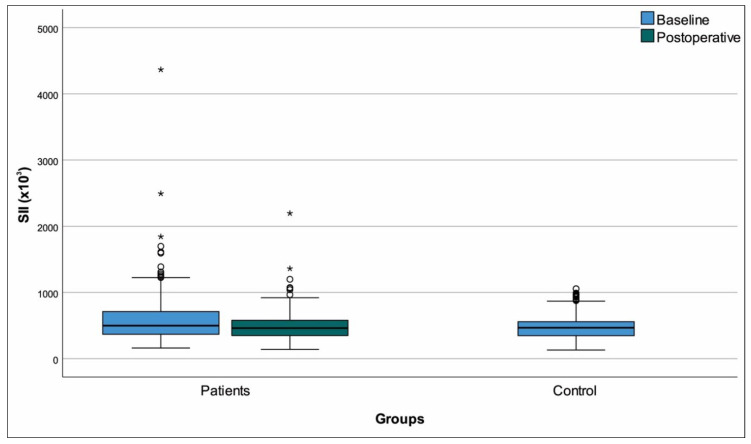
Box-plots systemic immune-inflammation index. “o Outliers, * Extreme outliers”.

**Figure 4:**
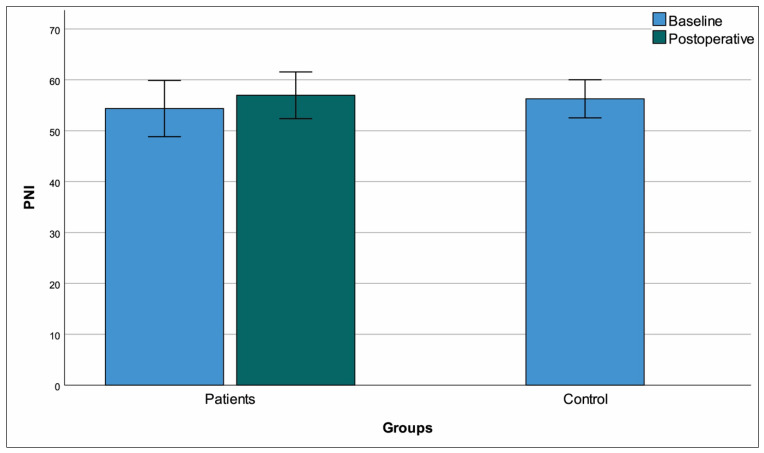
Mean ± standard deviation plots of prognostic nutritional index.

Females were overrepresented in the patient group compared to controls (*p* = 0.016) and the patient group was also older (*p* < 0.001). Comparisons to controls based on the baseline data of the entire patient group revealed multiple differences that were anticipated (Table 3). Among parameters that were significant at baseline comparison, the differences from controls persisted in the postoperative period for monocytes, hemoglobin, triglycerides, HDL-C, and MHR (Figure 2) (*p* < 0.001 for all). We also detected that postoperative results for calcium, albumin, CRP, total cholesterol SII (Figure 3), and PNI (Figure 4) had returned to levels that were similar to healthy controls (*p* > 0.05 for all). Of note, the longitudinally significant increase in platelet count (as shown in Table 2), appeared to have caused the appearance of a significant difference in the postoperative comparison against healthy controls (*p* = 0.003) (Table 3).

We performed propensity score matching due to the considerable differences between the groups in terms of age and sex, which yielded 259 patients matched for these characteristics in each of the patient and control groups. The statistical outcomes of comparisons between the groups remained largely similar; however, other significant differences from healthy controls emerged, particularly at baseline, including higher WBC (*p* = 0.006), higher neutrophil (*p* = 0.002), and higher platelet count (*p* = 0.014) (Table 4).

There were no significant differences for baseline and postoperative levels of inflammatory markers with regard to PHPT complications (nephrolithiasis and osteoporosis, Table 5 and Table 6, respectively).

Postoperative hypocalcemia was detected in 93 (23.48%) patients. Multivariable logistic regression analysis results had revealed that high baseline PTH (OR: 1.003, 95% CI: 1.001–1.005, *p* < 0.001) and low baseline total cholesterol (OR: 0.987, 95% CI: 0.979–0.995, *p* = 0.002) were independently associated with postoperative hypocalcemia (Table 7).

Postoperative SII was negatively correlated with baseline urine calcium (r = −0.136, *p* = 0.047). Postoperative PNI was negatively correlated with age at diagnosis (r = −0.265, *p* < 0.001), current age (r = −0.322, *p* < 0.001), and duration of disease (r = −0.238, *p* < 0.001) and was positively correlated with urine calcium (r = 0.215, *p* = 0.002). Postoperative MHR was positively correlated with male sex (r = 0.172, *p* = 0.007), duration of disease (r = 0.134, *p* = 0.034), baseline creatinine (r = 0.190, *p* = 0.003), and baseline ALP (r = 0.141, *p* = 0.040, Table 8).

## 4. Discussion

Parathyroid disorders disrupt calcium and phosphate balance as a result of abnormal PTH levels and are well established to influence immune function [21,22]. Our large cohort of patients who underwent PTX for PHPT demonstrated anticipated deviations from healthy controls at baseline and also many parameters showed a return to typical levels after treatment. Recent studies have reported not only the direct positive effects of PTX on calcium metabolism disturbances caused by hyperparathyroidism, but also its indirect effects, including blood pressure regulation [23], improvement in left ventricular hypertrophy [24], reduction in cardiovascular mortality [25], improvement in endothelial, vascular, and cardiac dysfunction [6,7], enhancement of quality of life [24], and positive effects on hematopoiesis [11,26]. Nonetheless, data are limited with respect to the effects of PTX on the inflammatory and nutritional changes that may arise from PHPT. Longitudinal analyses showed significant reductions in leukocyte counts, CRP, and SII following treatment, revealing a considerable reduction in systemic inflammation. In addition, albumin and PNI were marginally but significantly increased, approaching the levels measured in healthy controls, indicating improved nutritional status after treatment. Our analyses also extended to propensity-matched comparisons between healthy controls and the baseline and postoperative values of patients with PHPT, which showed that postoperative values returned to being similar to healthy control data for many crucial variables, including calcium, albumin, WBC, neutrophils, CRP, SII, and PNI.

Molecular evidence shows that PTH receptors are present in various immune cells and calcium signaling is established to facilitate immune response [21,22]; however, laboratory studies often fail to capture the complexity of clinical disease [27,28]. This is largely associated with the importance of metabolic balance and hormonal characteristics in immune response [29]. For instance, both hypo- and hyperparathyroidism have been associated with weakened immune defenses [21]. Calcium and phosphate imbalances can exacerbate the severity of infectious diseases [22]. As central players in various pathways and hormonal balance, the thyroid and parathyroid hormones are evidently crucial in inflammation and immunity. In patients with PHPT, a significant increase in the activity of inflammation-related genes has been reported [4]. Similarly, in animal models of hyperparathyroidism, alterations in gene expression and cytokine secretion have been observed [30]. Simpson et al. demonstrated that antigen stimulation in mast cells led to enhanced mediator release following exposure to PTH (1–34) [31]. Klinger et al. reported that PTH stimulated T-lymphocyte proliferation and increased cAMP production [32]. Adding to the complexity of these relationships, supraphysiological PTH concentrations are suggested to suppress lymphocyte functions [33]. The immunomodulatory effects of PTH through these pathways may explain the inflammatory phenotype observed in PHPT. Supraphysiological PTH levels likely induce a pro-inflammatory state via calcium-mediated immune cell activation (e.g., mast cell degranulation, T-cell proliferation) while simultaneously exhausting lymphocyte reserves through chronic stimulation [33]. PTX, by normalizing PTH levels, could reverse this dual effect: (1) reducing calcium-driven innate immune activation (neutrophils, CRP), and (2) restoring adaptive immune capacity (lymphocyte recovery). This mechanistic framework aligns with our findings of post-PTX reductions in SII (neutrophil↓/lymphocyte↑) and CRP, suggesting resolution of both chronic inflammation and immune exhaustion.

Despite these well-described impacts of hyperparathyroidism on inflammation, the restorative effect of PTX on inflammatory markers in patients with PHPT is a less-studied topic. As mentioned before, we found that WBC, neutrophil count, CRP, and SII all demonstrated significant reductions following PTX. Furthermore, the majority of significant inflammation-related differences between propensity-matched controls and patients at baseline (WBC, neutrophil, CRP, and SII) disappeared in the postoperative period, with monocyte count arguably being the only inflammation-related parameter that remained persistently higher among patients with PHPT. Deniz et al. reported that successful PTX reduces systemic inflammation in PHPT patients, with significant postoperative changes observed in inflammatory indices, including reductions in SII, platelet-to-lymphocyte ratio (PLR), and platelet distribution width (PDW), while an increase was noted in the monocyte-to-high-density lipoprotein cholesterol ratio. Logistic regression analysis identified PDW and PLR as significant markers of inflammation [5]. Unlike the study by Deniz et al., our investigation used a larger cohort, included matched healthy controls, and examined both SII and PNI in pre- and postoperative periods with propensity score analysis. Yang and colleagues also reported significant reductions in PLR and NLR following surgery [12]. In a retrospective study analyzing data from 95 patients diagnosed with PHPT who underwent PTX, a positive correlation was found between preoperative neutrophil-to-lymphocyte ratio (NLR) and calcium and PTH levels. Following curative PTX, the median NLR value significantly decreased [28], and others have shown postoperative reductions in lymphocyte count [26]. Another study focused on secondary HPT (SHPT) reported significant postoperative reductions in serum CRP, interleukin-6 (IL-6), and tumor necrosis factor-alpha (TNF-α) [13]. Similarly, Sato et al. found a longitudinal decrease in fibroblast growth factor-23 (FGF-23) levels, which aligned with reductions in serum phosphorus and calcium-phosphorus (Ca × P) product levels [34], which are results that have been replicated by a prospective study [35].

Nonetheless, there exist studies that do not show any notable changes in inflammation-related parameters following PTX; however, these are quite rare [36]. Recent evidence further suggests that postoperative increases in vitamin D levels may contribute to inflammation resolution in PHPT. As vitamin D deficiency is common in PHPT due to PTH-mediated catabolism, its surgical correction could potentially enhance the anti-inflammatory effects of PTX through vitamin D’s immunomodulatory properties, including suppression of pro-inflammatory cytokines and promotion of immune tolerance. This mechanism may act synergistically with direct PTH reduction to improve inflammatory markers [5]. The findings from our large cohort and propensity-matched comparisons indicate that PTX plays a crucial role in restoring typical systemic inflammation characteristics in patients with PHPT. The significant reductions in leukocytes, CRP, and SII emphasize the systemic benefits of PTX beyond calcium and hormonal balance. Notably, the decrease in SII suggests that PTX not only reduces neutrophil-driven inflammation but also restores immune balance by increasing lymphocyte counts. It is, therefore, conceivable that SII may serve as a tool for monitoring inflammatory changes in PHPT patients before and after surgery.

In relation to its strong impact on metabolic functions, PTH may also be associated with malnutrition in patients with PHPT, SHPT, and even tertiary hyperparathyroidism. There is substantial evidence suggesting that PTH significantly contributes to the pathogenesis of malnutrition [26,37]. For instance, elevated PTH has been associated with muscle wasting, weight loss, weakness, and negative nitrogen balance, all of which impair nutritional status [37]. These effects are believed to result from PTH’s impact on protein metabolism and skeletal muscle bioenergetics [37]. Studies have demonstrated that PTH disrupts energy production, transfer, and utilization in skeletal muscle [37], while also promoting muscle proteolysis and increasing the release of amino acids such as alanine and glutamine [27]. This acceleration in protein catabolism can deplete functional pathways, energy reserves and production, ultimately having the potential to exacerbate malnutrition. Indeed, carrying out PTX in patients with severe SHPT has been demonstrated to improve nutritional markers [38,39].

In our study, we observed increased albumin and PNI values following surgery in PHPT patients. Preoperatively, the albumin levels and PNI scores of the PHPT group were significantly lower than those of the control group; however, these differences disappeared postoperatively. Kir et al. [38,39] investigated tumor-derived PTH-related protein (PTHrP) and PTH in cachexia. They revealed that PTHrP increased thermogenic gene expression in adipose tissue, leading to muscle and fat loss. Similarly, PTH was found to indirectly trigger muscle loss by affecting adipose tissue in cachexia models associated with CKD and cancer. Loss of PTH receptors in adipose tissue was shown to prevent both fat and muscle loss, improving muscle mass and strength [38,39]. In a study longitudinally examining patients with hemodialysis, in was found that PTX resulted in significant weight gain during follow-up studies at 1, 3, 6, and 12 months after surgery. In fact, 53% of the patients experienced more than 5% increase in weight at 12 months [36]. Post-PTX improvements in weight, muscle mass, and nutrition-related measures have been collaborated by multiple studies involving patients with different baseline characteristics [10,13,40,41]. Despite strong evidence, it must be noted that there exist studies that have not detected any significant changes in PNI scores following surgery in PHPT patients [5], which might be associated with the limited sample size.

We additionally investigated potential associations between inflammatory markers (SII, PNI, MHR) and PHPT complications. We found no significant association between inflammatory markers (SII, PNI, MHR) and PHPT complications (nephrolithiasis and osteoporosis). These findings suggest that systemic inflammation may have a limited role in the pathophysiology of bone and renal complications in PHPT. Previous studies have similarly emphasized that bone loss and nephrolithiasis in PHPT are primarily associated with the direct effects of parathyroid hormone (PTH) and local mechanisms [42,43].

Regarding postoperative hypocalcemia, our analysis identified high baseline PTH levels and low total cholesterol as independent predictors. These results align with the hypothesis that severe hyperparathyroidism may increase hypocalcemia risk postoperatively due to the ‘hungry bone’ effect [44]. Furthermore, the association between low total cholesterol and hypocalcemia may indicate complex interactions between lipid metabolism and calcium homeostasis in PHPT. However, further studies are needed to elucidate the underlying mechanisms of this relationship.

Our findings align with the existing literature, demonstrating that PHPT patients exhibit significant improvements in albumin levels and PNI scores following surgery. The resolution of preoperative differences between the PHPT and control groups suggests that the metabolic disturbances induced by PTH are at least partially reversible. While this study focused on PHPT, the parallels with SHPT suggest that hyperparathyroidism, regardless of its etiology, plays a significant role in metabolic dysregulation and malnutrition. Additionally, while PTX appears to reverse many PTH-induced metabolic abnormalities, its long-term effects on body composition and energy metabolism require further investigation.

To our knowledge, this is the first study to comprehensively analyze both pre- and post-PTX alterations in laboratory variables in PHPT patients while also comparing these changes with a control group. Propensity matching from such a large original population also allowed for the alleviation of bias associated with age and sex. Nonetheless, several limitations should be acknowledged. The retrospective nature of the study inherently carries the risk of selection bias and confounding factors. This study primarily focused on laboratory parameters, without incorporating clinical and anthropometric measurements, which could provide a more comprehensive assessment of patient outcomes. Another limitation pertains to our postoperative follow-up timeline. The decision to analyze laboratory values obtained at least six months after PTX was based on the clinical rationale that this duration allows for the stabilization of calcium-PTH axis and resolution of acute postoperative inflammatory changes. However, the absence of intermediate time points (e.g., 1, 3, or 12 months) restricts our ability to delineate the dynamic trajectory of inflammatory and nutritional marker recovery. Early postoperative fluctuations—such as transient hypocalcemia or stress-induced leukocytosis—may have obscured short-term trends, while longer-term effects beyond six months (e.g., sustained nutritional improvements or late inflammatory rebound) remain unexplored. Future studies incorporating serial measurements would better characterize the temporal patterns of metabolic and immune recovery following PTX.

## 5. Conclusions

This study provides comprehensive evidence that PTX in PHPT patients leads to significant improvements in both systemic inflammation and nutritional status. Our key findings demonstrate: (1) marked reductions in leukocyte count, CRP, and SII; (2) significant increases in albumin and PNI, with postoperative values normalizing to healthy control levels; and (3) SII dynamics suggesting concurrent resolution of neutrophil-driven inflammation and lymphocyte recovery. These results are clinically important because they establish PTX as a treatment that addresses not just calcium metabolism but also PTH-induced metabolic and immune dysfunction. The SII findings particularly suggest its potential utility as a postoperative monitoring biomarker. This study elucidates the dual role of PTH in driving both inflammatory and nutritional disturbances, potentially identifying new therapeutic targets for non-surgical candidates. Future studies should validate these findings and investigate the long-term metabolic effects of PTX.

## Figures and Tables

**Table 1:** Demographics and disease-related characteristics.

Sex	
Female	333 (80.24%)
Male	82 (19.76%)
Age at diagnosis, years	53.40 ± 12.77
Current age, years	57.81 ± 13.31
Duration of disease, years	3 (1–7)
Adenoma location	
No adenoma	10 (2.88%)
Right inferior	156 (44.96%)
Right superior	19 (5.48%)
Left inferior	141 (40.63%)
Left superior	13 (3.75%)
Right intrathyroidal	2 (0.58%)
Left intrathyroidal	1 (0.29%)
Mediastinal	5 (1.44%)
Phosphorus	2.67 ± 0.67
PTH	178.5 (131.5–261.0)
25(OH)D3	13 (8–21)
Creatinine	0.7 (0.6–0.8)
ALP	93 (74–119)
Urine calcium	335 (242–471)
Normal	99 (39.13%)
Increased	154 (60.87%)
Nephrolithiasis	
Absent	149 (78.42%)
Present	41 (21.58%)
Bone mineral density, t score	
Femoral	−1.12 ± 1.09
Forearm	−2.35 ± 1.84
Lumbar spine	−1.57 ± 1.41
Osteoporosis	
Absent	95 (70.90%)
Present	39 (29.10%)
Final status	
Alive	413 (99.52%)
Exitus	2 (0.48%)

Descriptive statistics are presented using the mean ± standard deviation for normally distributed continuous variables, median (25th percentile–75th percentile) for non-normally distributed continuous variables, and frequency (percentage) for categorical variables. Abbreviations: 25(OH)D3: 25-hydroxyvitamin D3, ALP: alkaline phosphatase, PTH: parathyroid hormone

**Table 2:** Baseline and postoperative laboratory measurements.

	Baseline	Postoperative	*p*
Calcium	11.52 ± 0.89	9.20 ± 0.77	**<0.001 ^†^**
Albumin	42.67 ± 3.72	44.81 ± 3.11	**<0.001 ^†^**
WBC (×10^3^)	7.31 ± 1.72	7.14 ± 1.61	**0.019 ^†^**
Neutrophil (×10^3^)	4.31 ± 1.34	4.02 ± 1.15	**<0.001 ^†^**
Lymphocyte (×10^3^)	2.27 ± 0.72	2.40 ± 0.65	**0.002 ^†^**
Monocyte (×10^3^)	0.50 (0.38–0.60)	0.50 (0.39–0.60)	0.545 ^‡^
Hemoglobin	13.49 ± 1.61	13.46 ± 1.68	0.904 ^†^
RDW	13.7 (12.9–14.7)	13.6 (12.9–14.6)	0.298 ^‡^
Platelet (×10^3^)	271.64 ± 77.93	280.03 ± 75.46	**0.038 ^†^**
CRP	3.20 (1.15–6.85)	2.10 (1.02–4.00)	**<0.001 ^‡^**
Total cholesterol	195.61 ± 42.66	204.15 ± 42.62	**<0.001 ^†^**
Triglyceride	144 (99.5–192)	158.5 (110–225)	**<0.001 ^‡^**
HDL-C	45.93 ± 13.26	46.85 ± 13.37	0.235 ^†^
LDL-C	118.02 ± 35.15	121.42 ± 34.32	**0.009 ^†^**
SII (×10^3^)	483.85 (361.00–670.30)	453.70 (349.66–575.83)	**<0.001 ^‡^**
PNI	54.05 ± 5.35	56.63 ± 4.81	**<0.001 ^†^**
MHR	10.00 (7.41–14.96)	10.35 (7.30–15.22)	0.417 ^‡^

Descriptive statistics are presented using the mean ± standard deviation for normally distributed continuous variables, and median (25th percentile–75th percentile) for non-normally distributed continuous variables. ^†^ Paired *t* test, ^‡^ Wilcoxon signed ranks test. Statistically significant *p*-values are shown in bold. Abbreviations: CRP: C-reactive protein, HDL-C: high-density lipoprotein cholesterol, LDL-C: low-density lipoprotein cholesterol, MHR: monocyte to high-density lipoprotein cholesterol ratio, PNI: Prognostic Nutritional Index, RDW: red cell distribution width, SII: Systemic Immune-Inflammation Index, WBC: white blood cell count.

**Table 3:** Sex, age, and laboratory measurements with regard to groups for all available individuals.

	Groups		
	Patients (*n* = 415)	Controls (*n* = 410)	*p*
Sex			
Female	333 (80.24%)	300 (73.17%)	**0.016 ^§^**
Male	82 (19.76%)	110 (26.83%)	
Current age, years	57.81 ± 13.31	48.29 ± 14.64	**<0.001 ^†^**
Phosphorus			
Baseline	2.67 ± 0.67	3.42 ± 0.51	**<0.001 ^†^**
PTH			
Baseline	178.5 (131.5–261.0)	54.3 (41.2–70.5)	**<0.001 ^‡^**
Calcium			
Baseline	11.52 ± 0.89	9.25 ± 0.29	**<0.001 ^†^**
Postoperative	9.20 ± 0.77	-	0.183 ^†^
Albumin			
Baseline	42.67 ± 3.72	44.89 ± 1.69	**<0.001 ^†^**
Postoperative	44.81 ± 3.11	-	0.639 ^†^
WBC (×10^3^)			
Baseline	7.31 ± 1.72	7.16 ± 1.63	0.189 ^†^
Postoperative	7.14 ± 1.61	-	0.873 ^†^
Neutrophil (×10^3^)			
Baseline	4.31 ± 1.34	4.15 ± 1.17	0.079 ^†^
Postoperative	4.02 ± 1.15	-	0.121 ^†^
Lymphocyte (×10^3^)			
Baseline	2.27 ± 0.72	2.37 ± 0.68	0.061 ^†^
Postoperative	2.40 ± 0.65	-	0.434 ^†^
Monocyte (×10^3^)			
Baseline	0.50 (0.38–0.60)	0.42 (0.36–0.52)	**<0.001 ^‡^**
Postoperative	0.50 (0.39–0.60)	-	**<0.001 ^‡^**
Hemoglobin			
Baseline	13.49 ± 1.61	13.96 ± 1.44	**<0.001 ^†^**
Postoperative	13.46 ± 1.68	-	**<0.001 ^†^**
RDW			
Baseline	13.7 (12.9–14.7)	13.5 (13.0–14.2)	0.362 ^‡^
Postoperative	13.6 (12.9–14.6)	-	0.189 ^‡^
Platelet (×10^3^)			
Baseline	271.64 ± 77.93	264.57 ± 60.06	0.145 ^†^
Postoperative	280.03 ± 75.46	-	**0.003 ^†^**
CRP			
Baseline	3.20 (1.15–6.85)	2.10 (1.10–3.70)	**<0.001 ^‡^**
Postoperative	2.10 (1.02–4.00)	-	0.357 ^‡^
Total cholesterol			
Baseline	195.61 ± 42.66	206.30 ± 45.37	**0.002 ^†^**
Postoperative	204.15 ± 42.62	-	0.534 ^†^
Triglyceride			
Baseline	144 (99.5–192)	128 (89–187)	**0.020 ^‡^**
Postoperative	158.5 (110–225)	-	**<0.001 ^‡^**
HDL-C			
Baseline	45.93 ± 13.26	55.40 ± 12.62	**<0.001 ^†^**
Postoperative	46.85 ± 13.37	-	**<0.001 ^†^**
LDL-C			
Baseline	118.02 ± 35.15	121.13 ± 35.84	0.262 ^†^
Postoperative	121.42 ± 34.32	-	0.914 ^†^
SII (×10^3^)			
Baseline	483.85 (361.00–670.30)	469.21 (361.67–580.63)	**0.034 ^‡^**
Postoperative	453.70 (349.66–575.83)	-	0.602 ^‡^
PNI			
Baseline	54.05 ± 5.35	56.72 ± 3.83	**<0.001 ^†^**
Postoperative	56.63 ± 4.81	-	0.782 ^†^
MHR			
Baseline	10.00 (7.41–14.96)	7.54 (5.97–10.36)	**<0.001 ^†^**
Postoperative	10.35 (7.30–15.22)	-	**<0.001 ^†^**

Descriptive statistics are presented using the mean ± standard deviation for normally distributed continuous variables, median (25th percentile–75th percentile) for non-normally distributed continuous variables, and frequency (percentage) for categorical variables. ^§^ Chi-square test, ^†^ Student’s *t* test, ^‡^ Mann–Whitney U test. Statistically significant *p* values are shown in bold. Abbreviations: CRP: C-reactive protein, HDL-C: high-density lipoprotein cholesterol, LDL-C: low-density lipoprotein cholesterol, MHR: monocyte to high-density lipoprotein cholesterol ratio, PNI: Prognostic Nutritional Index, RDW: red cell distribution width, SII: Systemic Immune-Inflammation Index, WBC: white blood cell count, PTH: parathyroid hormone.

**Table 4:** Sex, age, and laboratory measurements with regard to groups for propensity score-matched (sex and age) individuals.

	Groups		
	Patients (*n* = 259)	Controls (*n* = 259)	*p*
Sex			
Female	202 (77.99%)	205 (79.15%)	0.748 ^§^
Male	57 (22.01%)	54 (20.85%)	
Current age, years	55.34 ± 12.51	53.56 ± 14.57	0.137 ^†^
Phosphorus			
Baseline	2.69 ± 0.62	3.48 ± 0.51	**<0.001 ^†^**
PTH			
Baseline	173.5 (130.5–248.5)	55.7 (42.6–72.6)	**<0.001 ^‡^**
Calcium			
Baseline	11.50 ± 0.88	9.26 ± 0.29	**<0.001 ^†^**
Postoperative	9.25 ± 0.75	-	0.830 ^†^
Albumin			
Baseline	43.00 ± 3.79	44.81 ± 1.56	**<0.001 ^†^**
Postoperative	45.00 ± 3.02	-	0.369 ^†^
WBC (×10^3^)			
Baseline	7.34 ± 1.76	6.94 ± 1.59	**0.006 ^†^**
Postoperative	7.12 ± 1.54	-	0.182 ^†^
Neutrophil (×10^3^)			
Baseline	4.34 ± 1.35	4.00 ± 1.12	**0.002 ^†^**
Postoperative	4.01 ± 1.11	-	0.951 ^†^
Lymphocyte (×10^3^)			
Baseline	2.27 ± 0.77	2.29 ± 0.68	0.743 ^†^
Postoperative	2.39 ± 0.63	-	0.085 ^†^
Monocyte (×10^3^)			
Baseline	0.50 (0.40–0.60)	0.42 (0.35–0.52)	**<0.001 ^‡^**
Postoperative	0.50 (0.40–0.60)	-	**<0.001 ^‡^**
Hemoglobin			
Baseline	13.48 ± 1.56	13.82 ± 1.35	**0.010 ^†^**
Postoperative	13.49 ± 1.70	-	**0.018 ^†^**
RDW			
Baseline	13.7 (12.9–14.9)	13.5 (13.0–14.1)	0.159 ^‡^
Postoperative	13.6 (13.0–14.6)	-	0.153 ^‡^
Platelet (×10^3^)			
Baseline	277.52 ± 80.89	262.05 ± 61.13	**0.014 ^†^**
Postoperative	281.33 ± 77.79	-	**0.002 ^†^**
CRP			
Baseline	3.2 (1.2–6.0)	2.0 (1.0–3.3)	**0.001 ^‡^**
Postoperative	2.0 (1.0–3.8)	-	0.557 ^‡^
Total cholesterol			
Baseline	198.78 ± 43.81	206.43 ± 48.84	0.085 ^†^
Postoperative	205.17 ± 44.09	-	0.776 ^†^
Triglyceride			
Baseline	146 (101–200)	104 (73–167)	**<0.001 ^‡^**
Postoperative	159 (110–225)	-	**<0.001 ^‡^**
HDL-C			
Baseline	46.56 ± 13.41	57.48 ± 12.76	**<0.001 ^†^**
Postoperative	47.48 ± 13.69	-	**<0.001 ^†^**
LDL-C			
Baseline	119.53 ± 35.55	121.71 ± 38.51	0.538 ^†^
Postoperative	121.86 ± 34.82	-	0.966 ^†^
SII (×10^3^)			
Baseline	497.91 (368.00–714.82)	467.34 (346.98–559.15)	**0.005 ^‡^**
Postoperative	460.95 (348.13–581.41)	-	0.772 ^‡^
PNI			
Baseline	54.36 ± 5.53	56.28 ± 3.74	**<0.001 ^†^**
Postoperative	56.97 ± 4.59	-	0.062 ^†^
MHR			
Baseline	10.14 (7.41–15.15)	7.06 (5.69–9.83)	**<0.001 ^†^**
Postoperative	10.00 (7.35–14.55)	-	**<0.001 ^†^**

Descriptive statistics are presented using the mean ± standard deviation for normally distributed continuous variables, median (25th percentile–75th percentile) for non-normally distributed continuous variables, and frequency (percentage) for categorical variables. ^§^ Chi-square test, ^†^ Student’s *t* test, ^‡^ Mann–Whitney U test. Statistically significant *p*-values are shown in bold. Abbreviations: CRP: C-reactive protein, HDL-C: high-density lipoprotein cholesterol, LDL-C: low-density lipoprotein cholesterol, MHR: monocyte to high-density lipoprotein cholesterol ratio, PNI: Prognostic Nutritional Index, RDW: red cell distribution width, SII: Systemic Immune-Inflammation Index, WBC: white blood cell count, PTH: parathyroid hormone.

**Table 5:** Summary of inflammatory markers with regard to nephrolithiasis in the patients.

	Nephrolithiasis		
	Absent (*n* = 149)	Present (*n* = 41)	*p*
SII (×10^3^)			
Baseline	481.10 (353.23–662.47)	512.24 (378.63–740.18)	0.354 ^‡^
Postoperative	440.00 (342.33–570.96)	455.62 (392.14–677.78)	0.169 ^‡^
PNI			
Baseline	54.31 ± 5.40	53.56 ± 4.83	0.424 ^†^
Postoperative	56.83 ± 4.89	56.31 ± 4.49	0.577 ^†^
MHR			
Baseline	10.00 (7.50–14.63)	12.22 (7.41–16.22)	0.409 ^‡^
Postoperative	9.52 (7.27–14.63)	11.93 (6.83–17.95)	0.436 ^‡^

Descriptive statistics are presented using the mean ± standard deviation for normally distributed continuous variables and the median (25th percentile–75th percentile) for non-normally distributed continuous variables. ^†^ Student’s *t* test, ^‡^ Mann–Whitney U test. Abbreviations: MHR: monocyte to high-density lipoprotein cholesterol ratio, PNI: Prognostic Nutritional Index, SII: Systemic Immune-Inflammation Index.

**Table 6:** Summary of inflammatory markers with regard to osteoporosis in the patients.

	Osteoporosis		
	Absent (*n* = 95)	Present (*n* = 39)	*p*
SII (×10^3^)			
Baseline	480.38 (361.00–714.82)	479.60 (385.25–725.59)	0.824 ^‡^
Postoperative	438.67 (351.93–571.27)	454.58 (347.14–601.66)	0.692 ^‡^
PNI			
Baseline	55.09 ± 5.41	54.91 ± 4.24	0.858 ^†^
Postoperative	57.57 ± 4.68	56.84 ± 4.96	0.450 ^†^
MHR			
Baseline	10.87 (7.58–16.00)	10.53 (6.52–14.63)	0.351 ^‡^
Postoperative	10.00 (6.98–15.00)	10.42 (7.84–16.67)	0.497 ^‡^

Descriptive statistics are presented using mean ± standard deviation for normally distributed continuous variables and the median (25th percentile–75th percentile) for non-normally distributed continuous variables. ^†^ Student’s *t* test, ^‡^ Mann–Whitney U test. Abbreviations: MHR: monocyte to high-density lipoprotein cholesterol ratio, PNI: Prognostic Nutritional Index, SII: Systemic Immune-Inflammation Index.

**Table 7:** Predictors of postoperative hypocalcemia, logistic regression analysis results.

	Univariable		Multivariable ^†^	
	OR (95% CI)	*p*	OR (95% CI)	*p*
Sex, Male	1.449 (0.833–2.520)	0.189		
Age at diagnosis, years	0.997 (0.979–1.016)	0.776		
Current age, years	1.005 (0.987–1.023)	0.604		
Duration of disease, years	1.103 (1.033–1.178)	**0.003**		0.324
Phosphorus, Baseline	0.803 (0.551–1.171)	0.254		
PTH, Baseline	1.002 (1.001–1.004)	**<0.001**	1.003 (1.001–1.005)	**<0.001**
25(OH)D3, Baseline	0.998 (0.965–1.032)	0.914		
Creatinine, Baseline	1.351 (0.620–2.944)	0.450		
ALP, Baseline	1.006 (1.002–1.010)	**0.003**		0.136
Calcium, Baseline	1.151 (0.896–1.478)	0.272		
Albumin, Baseline	0.895 (0.841–0.952)	**<0.001**		0.412
WBC (×10^3^), Baseline	0.833 (0.722–0.962)	**0.013**		0.284
Neutrophil (×10^3^), Baseline	0.915 (0.767–1.092)	0.325		
Lymphocyte (×10^3^), Baseline	0.567 (0.397–0.809)	**0.002**		0.281
Monocyte (×10^3^), Baseline	0.290 (0.082–1.025)	0.055		
Hemoglobin, Baseline	0.894 (0.772–1.036)	0.136		
RDW, Baseline	0.974 (0.876–1.083)	0.631		
Platelet (×10^3^), Baseline	0.997 (0.994–1.000)	0.081		
CRP, Baseline	1.028 (0.952–1.110)	0.480		
Total cholesterol, Baseline	0.987 (0.979–0.995)	**0.001**	0.987 (0.979–0.995)	**0.002**
Triglyceride, Baseline	0.997 (0.993–1.001)	0.158		
HDL-C, Baseline	0.983 (0.961–1.006)	0.149		
LDL-C, Baseline	0.987 (0.978–0.996)	**0.005**		0.286
SII (×10^3^), Baseline	1.000 (1.000–1.001)	0.600		
PNI, Baseline	0.897 (0.856–0.940)	**<0.001**		0.183
MHR, Baseline	0.990 (0.947–1.036)	0.675		
Urine calcium, Baseline	1.001 (1.000–1.003)	0.067		
Nephrolithiasis, Present	1.293 (0.581–2.875)	0.529		
Osteoporosis, Present	1.060 (0.418–2.690)	0.903		
Nagelkerke R^2^	-		0.162	

^†^ Multivariable analysis was performed via forward conditional selection method. Statistically significant *p*-values are shown in bold. Abbreviations: 25(OH)D3: 25-hydroxyvitamin D3, ALP: alkaline phosphatase, CI: confidence interval, CRP: C-reactive protein, HDL-C: high-density lipoprotein cholesterol, LDL-C: low-density lipoprotein cholesterol, MHR: monocyte to high-density lipoprotein cholesterol ratio, OR: odds ratio, PNI: Prognostic Nutritional Index, RDW: red cell distribution width, SII: Systemic Immune-Inflammation Index, WBC: white blood cell count, PTH: parathyroid hormone.

**Table 8:** Correlations between postoperative SII, PNI, and MHR levels and disease-related characteristics.

		SII (×10^3^)	PNI	MHR
Sex, Male	r	0.049	−0.034	0.172
	*p*	0.372	0.543	**0.007**
Age at diagnosis, years	r	0.015	−0.265	0.057
	*p*	0.787	**<0.001**	0.367
Current age, years	r	0.030	−0.322	0.098
	*p*	0.592	**<0.001**	0.121
Duration of disease, years	r	0.054	−0.238	0.134
	*p*	0.329	**<0.001**	**0.034**
Phosphorus, Baseline	r	−0.049	0.106	−0.020
	*p*	0.384	0.057	0.752
PTH, Baseline	r	0.025	−0.077	−0.055
	*p*	0.666	0.180	0.399
25(OH)D3, Baseline	r	−0.024	−0.020	−0.010
	*p*	0.729	0.771	0.898
Creatinine, Baseline	r	0.069	−0.100	0.190
	*p*	0.218	0.073	**0.003**
ALP, Baseline	r	−0.030	0.022	0.141
	*p*	0.623	0.721	**0.040**
Calcium, Baseline	r	0.028	0.004	0.006
	*p*	0.622	0.947	0.931
Urine calcium, Baseline	r	−0.136	0.215	−0.058
	*p*	**0.047**	**0.002**	0.453
Nephrolithiasis, Present	r	0.109	−0.044	0.068
	*p*	0.169	0.577	0.438
Osteoporosis, Present	r	0.037	−0.070	0.069
	*p*	0.694	0.450	0.499

r: Correlation coefficient, Statistically significant *p*-values are shown in bold. Abbreviations: 25(OH)D3: 25-hydroxyvitamin D3, ALP: alkaline phosphatase, MHR: monocyte to high-density lipoprotein cholesterol ratio, PNI: Prognostic Nutritional Index, SII: Systemic Immune-Inflammation Index, PTH: parathyroid hormone.

## Data Availability

The datasets generated during and/or analyzed during the current study are not publicly available but are available from the corresponding author on reasonable request.

## Abbreviations

ALP, alkaline phosphatase; BMD, bone mineral density; CRP, C-reactive protein; HDL-C, high-density lipoprotein cholesterol; LDL-C, low-density lipoprotein cholesterol; NLR, neutrophil-to-lymphocyte ratio; PDW, platelet distribution width; PHPT, primary hyperparathyroidism; PLR, body mass index; PNI, Prognostic Nutritional Index; PTH, parathyroid hormone; PTX, parathyroidectomy; RDW, red cell distribution width; TNF-α, tumor necrosis factor-alpha; SII, Systemic Immune-Inflammation Index; WBC, white blood cell.
